# Evaluation of a Clinical Service Model for Dysphagia Assessment via Telerehabilitation

**DOI:** 10.1155/2013/918526

**Published:** 2013-12-08

**Authors:** Elizabeth C. Ward, Clare L. Burns, Deborah G. Theodoros, Trevor G. Russell

**Affiliations:** ^1^The University of Queensland, School of Health & Rehabilitation Sciences, St. Lucia, Brisbane, QLD 4072, Australia; ^2^Centre for Functioning and Health Research, Queensland Health, Buranda, Brisbane, QLD 4102, Australia; ^3^Speech Pathology Department, Royal Brisbane and Women's Hospital, Herston, Brisbane, QLD 4006, Australia

## Abstract

Emerging research supports the feasibility and viability of conducting clinical swallow examinations (CSE) for patients with dysphagia via telerehabilitation. However, minimal data has been reported to date regarding the implementation of such services within the clinical setting or the user perceptions of this type of clinical service. A mixed methods study design was employed to examine the outcomes of a weekly dysphagia assessment clinic conducted via telerehabilitation and examine issues relating to service delivery and user perceptions. Data was collected across a total of 100 patient assessments. Information relating to primary patient outcomes, session statistics, patient perceptions, and clinician perceptions was examined. Results revealed that session durations averaged 45 minutes, there was minimal technical difficulty experienced, and clinical decisions made regarding primary patient outcomes were comparable between the online and face to face clinicians. Patient satisfaction was high and clinicians felt that they developed good rapport, found the system easy to use, and were satisfied with the service in over 90% of the assessments conducted. Key factors relating to screening patient suitability, having good general organization, and skilled staff were identified as facilitators for the service. This trial has highlighted important issues for consideration when planning or implementing a telerehabilitation service for dysphagia management.

## 1. Introduction

There is a small but emerging evidence base supporting the use of telerehabilitation to improve access to both clinical and instrumental dysphagia assessment services [1–8]. However, while the research conducted to date has focused on the evaluation of different types of system architecture [1, 4, 6], early feasibility data [1, 4, 7, 8], and the validity and reliability of online clinical decisions [2, 3, 5, 7, 8], no studies have examined the service characteristics (session durations, session complication rates, and equipment issues) associated with implementing a telerehabilitation clinical service. There is also limited information regarding potential facilitators and barriers to implementing a successful and time efficient telerehabilitation dysphagia service. Only one paper to date has discussed issues noted during the assessment of a small set of patients where certain patient factors (e.g., hearing impairment, movement disorders) made the assessment process less efficient [9]. That paper highlighted important service considerations including the need for careful patient and clinician preparation prior to the session, having flexible system capabilities (e.g., multiple adjustable cameras, free field and lapel microphones, etc.) which allow for modifications/adjustments to assist the patient, and the importance of good support staff at the patient end to facilitate the session.

Integral to the evaluation of any new clinical service is also the examination of the consumer perspective. Without consumer support, from both the clinicians providing the services and the patients receiving them, new service models will not be adopted or sustained. Recent data from a cohort of 40 patients has shown that both clinician and patient satisfaction with dysphagia assessments provided via telerehabilitation were high [2]. However, although patient perceptions were positive, use of a pre- and postsession methodology revealed that a small proportion of patients had some presession reservations, stemming from a lack of awareness/understanding of what a telerehabilitation session would be like. Patient concerns about the “unknown” telerehabilitation service may have implications for the uptake of services.

Clinical service information is critical to inform the next step of telerehabilitation service planning and enable the incorporation of telerehabilitation services as a successful and viable service model for patients with dysphagia. The aim of the current research is to evaluate a short-term trial of a telerehabilitation service providing clinical assessments of dysphagia. By examining the service characteristics, barriers, facilitators, and the consumer perspective, the study aims to define the scope of the strengths and challenges involved with implementing this new service model.

## 2. Materials and Methods

A total of 100 patients being managed by the speech pathology department of the Royal Brisbane and Women's Hospital, QLD, Australia, were recruited to receive a clinical swallow examination via a weekly telerehabilitation dysphagia assessment clinic. Participants were recruited across a range of dysphagia severity levels (normal, mild, moderate, and severe) ensuring diversity within the sample. For recruitment, dysphagia severity was determined from a Clinical Swallow Examination (CSE) conducted by a clinician independent of this research, at a time no greater than 24 hours prior to the online clinic assessment. From the findings of that CSE, dysphagia severity was classified using the 7-level Dysphagia Outcome and Severity Scale [10] (nondysphagic: level 6-7; mild: level 5; moderate: level 3-4, severe: level 1-2). For inclusion, participants had to be deemed suitable for assessment by their treating medical officer and capable of remaining in a semiupright or upright position for the duration of the assessment. They were not required to have any knowledge or skills associated with computers or technology.

The participant group recruited was 54% male with a mean age of 67.08 years (SD = 16.99, range 21–112). One-quarter had a medically diagnosed cognitive deficit. Participants were from a range of aetiological groups (51% acute/degenerative disorder; 31% cancer care; 18% other) and presented with nil to severe dysphagia (25 nondysphagic; 25 mild, 25 moderate, and 25 severe). Consent was obtained for all participants. The study was granted ethical clearance from the Human Research Ethics Committees of both Queensland Health and The University of Queensland.

### 2.1. The Telerehabilitation Clinic

The administrative, clinical, and technical guidelines for telerehabilitation practice [11] were used to establish the clinic and its processes. The trial clinic ran for 4 hours on a weekday morning with a maximum of 4 patients scheduled into any clinic. Clinic staff on any day included an online speech pathologist (O-SP) who arranged and led all assessments and an allied health assistant located at the patient end who was responsible for arranging inpatient transport to the telerehabilitation clinic, setting up the system, positioning and preparing the patient, and assisting with physical tasks (e.g., feeding the patient during the food and fluid trials) under the direction of the O-SP. A second face to face speech pathologist (FTF-SP) was also involved in each clinic and was located in the room with the patient. The role of the FTF-SP was to conduct a simultaneous CSE (with the O-SP) in the FTF environment for each patient. This allowed later comparison of the online and FTF assessment results to calculate clinical agreement. The two SPs involved in any session (online or FTF) came from a pool of four clinicians, each with >5 years of experience in managing dysphagia in the acute care setting. For research purposes, distance was simulated, with the O-SP located in one room of the clinical setting and the patient, assistant, and FTF-SP located in a separate room within the same facility.

### 2.2. The Telerehabilitation System

The telerehabilitation system used in this study has been described in detail elsewhere [1–3]. It consisted of notebook computers at the patient and clinician ends that incorporated custom video conferencing software audio and video compression technology for real-time videoconferencing. Audio was enabled using a free-field combined echo cancelling microphone and web-conference speaker, while the patient also wore a lapel microphone. Fixed and free standing cameras (with zoom capacity) were incorporated and remotely controlled by the O-SP. The system enabled the capture of audio and video (640 × 480 pixels) at the client end of the consultation for store-and-forward recordings of the sessions. An ad hoc 802.11 g wireless network with a throttled bandwidth of 128 Kbit/s was used for communication. This low bandwidth was purposefully chosen as it is the minimum bandwidth available across Australia's public health network.

### 2.3. Clinical Swallow Examination (CSE)

The CSE procedure has been reported in detail previously [1–3]. Prior to the session, both the online and FTF clinicians received a summary of the patient's relevant medical history. Each assessment was then led by the O-SP who based their clinical judgments on their online observations and/or on later review of the store and forward videos. The CSE followed a structured pro forma of 65 test items divided into four main sections including (1) general orientation and alertness, (2) oromotor and laryngeal function assessment, (3) performance during food and fluid trials, and (4) clinical decisions and recommendations.

### 2.4. Data Collection

The study employed a mix methods design to examine patient outcomes, session statistics, and patient and clinician perceptions. Patient outcome data was collected from the simultaneous CSEs conducted by both the O-SP and FTF-SPs during the clinic sessions. For this study, data from only 3 key patient outcome parameters collected as part of the “Clinical Decisions and Recommendations” component of the CSE were examined: (a) decision regarding safety for oral/nonoral feeding (binary decision), (b) recommended safe fluid intake (4-level categorical data), and (c) recommended safe food intake (4-level categorical data). In addition, data regarding the patients need for review/ongoing care was collated. For this study, this data was coded into 4 categories: review needed within 1 week; review within >1 week and <1 month; review in 3 months; or, patient can be discharged. Details of the levels of agreement for the full range of CSE items have been outlined elsewhere [3].

Session statistics were calculated from the session logs recorded by the videoconferencing system software. The system recorded the number of dropped connections/reconnections during any session. It also recorded the total time (in minutes) of the online session. The timing data related only to the duration of the online assessment and did not include time spent by the assistant preparing the room/patient or the time spent by the clinician reading the medical history or writing their report. Comments pertaining to any equipment failure or visual or auditory difficulties were collected through postsession reports completed by the O-SP.

Patient perceptions were explored using a questionnaire delivered both before and after the telerehabilitation session as per prior research [12]. This contained 14 items that examined perceptions regarding (1) level of comfort with telerehabilitation (3 questions), (2) audio and video quality (2 questions), and (3) general considerations regarding telerehabilitation consultation (9 questions) (detailed in Table 1). The questions in the pre- and postquestionnaire were matched to explore patient perceptions before and after the telerehabilitation session (e.g., before: “*I will have* no difficulty seeing the online speech pathologist”; after: “I *had* no difficulty seeing the online speech pathologist”). Responses were rated using a five-point scale (1: strongly disagree, 2: disagree, 3: unsure, 4: agree, 5: strongly agree).

Perceptions of the clinicians were explored through (a) a satisfaction questionnaire completed at the end of every session and (b) a single semistructured interview completed once at the end of testing the 100 patients. The satisfaction questionnaire has been published previously [2] and addressed (1) satisfactions with the system (4 questions), (2) the perceived level of patient-clinician rapport (1 question), (3) satisfaction with the level of service provided to the patient (1 question), and (4) suitability of a telerehabilitation assessment for the individual patient (2 questions) (detailed in Table 2). Satisfaction was rated on a five-point scale (1: strongly disagree, 3: unsure, 5: strongly agree). The semistructured clinician interview took approximately 15 minutes and involved reflecting on elements which helped make the clinic successful/unsuccessful.

### 2.5. Data Analysis

Levels of exact agreement between the O-SP and FTF-SP decisions for the three patient outcomes were calculated using percentages. A level of ≥80% exact agreement (for nominal/categorical data) was used to represent clinically acceptable levels of agreement, as per prior research [1–5, 13]. Session statistics were analysed descriptively. Patient perceptions before and after the session were collapsed into 3 groups (disagree/strongly disagree, unsure, and agree/strongly agree) and are reported descriptively. Comparison between proportion change over time was analysed using Chi-square or Fisher's exact test. Significance was set at *P* < 0.05. Results of the postsession clinician satisfaction questionnaires were compiled descriptively. The content of the semistructured clinician interview was interpreted live by the interviewer who made notes during the interview. At the end of the interview, the interviewer summarized the key points and checked the accuracy of these with the interviewee.

## 3. Results

Analysis of patient outcomes revealed that 100% of sessions reached a clinical decision regarding patient intake status. Level of PEA between the O-SP and FTF-SP was 99% for the decision to place the patient oral or nonoral. For the 1 patient where the oral/nonoral decision was in disagreement, the FTF clinician had placed the patient completely on nonoral intake, while the teleclinician had essentially made the same decision, though they had allowed the person small sips of thickened fluids under speech pathology supervision in addition to nonoral supplementation. Level of agreement for safe fluid and food consistencies was 98% and 92%, respectively. For the 2 disagreements for fluid ratings and the 8 for food consistency ratings, these differed by no more than one fluid/diet level and no decision could be considered unsafe. Regarding the need for review/ongoing management, there was 88% exact agreement. Overall the O-SP recommended that 66 patients should be reviewed again within 1 week (to check safety on recommended diet or for ongoing reassessment), 27 in >1 week but <1 month, 1 in 3 months, and 6 were discharged. All incidences of disagreement were examined to explore any potential bias for either the O-SP or FTF-SP ratings. There was no clear pattern observed to support more conservative decision making or a particular pattern of error/disagreement occurring in either environment.

Session statistics revealed an average duration of 45 minutes (SD: 13, mode: 46, range 22–80). The first quartile of the cohort (i.e., the shortest times) ranged from 22 to 37 minutes and contained 80% normal or mildly dysphagic patients. The top 25% (i.e., longest session durations) ranged between 48 and 80 minutes and contained 68% moderate or severe patients. Disconnections were rare, occurring in only 10% of the sessions with the maximum number of disconnections in any session being 2. However, clinicians reported difficulties at times during sessions with periods of reduced audio (long delays) and/or visual quality (heavy pixilation) in 22% of the sessions. These audio and visual quality issues were identified as difficulties by clinicians; however, they did not prevent successful completion of the assessment. Only 6 assessments were cancelled outright and rescheduled due to equipment issues (camera frozen and not enabling zoom capabilities; system audio not functional), though trouble shooting and/or replacement equipment enabled subsequent patient assessments within the scheduled clinic to be completed.

Although all 100 patients were successfully assessed in the clinic, eighteen participants were cognitively unable to complete the patient questionnaires before or after the session. Examination of the questionnaire data from the other 82 revealed that greater than two-thirds agreed/strongly agreed with most questions before the session (Table 1). However, there were some patients who were unsure or disagreed with the questions relating to being able to see and hear the online clinician, if they believed instructions would be clear, if they would have sufficient time to execute tasks, or if the online assessment would be equal to or able to replace face to face sessions. There were also over 77% of patients who indicated that they were either unsure or agreed that they would prefer a traditional assessment in their presession questionnaire.

Patient perceptions immediately following the telerehabilitation session, however, revealed many of these negative positions had changed significantly (Table 1). A significant shift from 37 to 80% of patients agreed they had no difficulty seeing the online clinician. A significant change from 57% to 83% felt the online assessment was equal to a FTF assessment after the session. Significantly more patients felt that telerehabilitation could replace a FTF session, allowing easy access to healthcare, and could benefit all patients. There was also a change regarding preference for a traditional assessment with significantly more patients disagreeing they would prefer a traditional assessment. However, 37% continued to agree that they would prefer a traditional assessment (Table 1). Although responses to other questions were not significantly different from the presession perceptions, patterns of nonsignificant shifts towards agreeing with all statements were noted.

The postsession questionnaires completed by the clinicians revealed that over 90% who agreed/strongly agreed were happy with the service they could provide via the system, their level of rapport with patients, the ease of use of the system, their ability to assess the patient, and the usefulness of telerehabilitation as a service delivery option (Table 2). In 18% of sessions the clinicians did not agree that the audio quality was appropriate and in 17% of sessions they felt the video image was not adequate. For 24% of sessions, clinicians either were unsure (12%) or disagreed (12%) that telerehabilitation would be a more efficient means of service delivery for that patient. Where they disagreed, these were sessions with patients with severely reduced vocal volume (making detecting clinical signs of aspiration difficult) and those with an inability to follow commands and instructions, excess body movements, significant hearing or vision impairment, fatigue, distress/agitation, and/or overall severe medical state.

The clinician interview allowed reflection on issues that had been problematic and factors which facilitated optimal functioning of the clinic. These fell into the 3 categories: patient considerations, general organisational issues, and staff roles. “Patient considerations” included multifactorial issues relating to the patients health and suitability for the clinic. Referrals need to be screened for suitability (i.e. to exclude patients with very low levels of alertness, or those with highly unstable medical states) prior to the clinic. Clinicians also noted the importance of ensuring that there are clear patient details and case history information provided to allow the online clinician and assistant to prepare for the session and to plan how to manage any patient factors (hearing impairment, cognitive deficits) which could raise challenges during the online assessment.

“General organisation” issues raised the importance of allowing 1 hour for each patient appointment to accommodate longer/more complex assessments and potential technology/connection issues. Preclinic setup (testing equipment, etc.) was seen as essential to the clinic running smoothly, as were advance bookings for porterage to transport inpatients to and from the telehealth room on time. Good communication between the booking site (patient end) and the hub site (O-SP end) to confirm appointments and any changes was critical. Clear protocols for referrals, appointments and session reporting at both the patient end and online clinician site needed to be established. Attendance at the clinic was also maximised by ensuring the appointment and patient status/suitability was re-confirmed the morning prior to the assessment clinic.

Clinicians noted that staff skills assisted the functioning of the clinic. The importance of having an assistant with good patient skills and manual handling skills was necessary for establishing rapport with the patient and repositioning during the session. They also commented that as they became more experienced, their ability to conduct the online session more efficiently improved. Convenient access to technical staff for equipment issues and ensuring that the staff involved in the clinic are trained in equipment operation and basic trouble shooting were also seen as important.

## 4. Discussion

The mixed methodology employed by the current study enabled examination of both the feasibility and consumer acceptance of a dysphagia telerehabilitation clinical service. Overall the data revealed a viable clinical service model which had good patient outcomes and minimal technical issues. Patient perceptions and clinician perceptions were also positive. For those clinicians who provide dysphagia services across multiple locations, or outreach services to rural locations, the current data supports the potential feasibility of using a telerehabilitation clinic model to enhance patient access to services.

Equipment and technical problems were minimal. The clinic adhered to published technical recommendations for telerehabilitation [11] and staff stressed that developing good technical trouble shooting skills and having access to technical staff were integral to the clinic's success. Only 6 individual assessment sessions needed to be cancelled and rescheduled due to equipment malfunction/technical issues. However, these technical issues were quickly rectified allowing continuation of the clinic and subsequent assessment sessions. Despite some reduced audio and/or visual quality at times during 22% of sessions, all assessments which were commenced were completed and a clinical decision regarding patient safety for oral intake was determined. It was not unexpected that some visual and auditory quality issues were experienced during sessions, as testing took place at intentionally controlled low bandwidths used to ensure the clinic's feasibility at the absolute minimum bandwidth available within the health network. Future service functionality has capacity to occur at much higher bandwidths. Hence, this will reduce the instances of audio delays and image pixillation experienced at low bandwidths.

Despite the presence of some slight reductions in visual or auditory quality, overall the quality of the clinical decisions between the O-SP and FTF-SP was found to be highly comparable, falling well above the 80% agreement criteria set in this study. This result is consistent with previous research [1–3] and supports the validity of clinical decisions made within the online environment. Where discrepancies occurred between the O-SP and FTF-SP, there was also no clear pattern observed to suggest that the online clinician made more conservative judgments or any particular pattern of errors in decision making. As such, the differences observed between the two raters appear to be best explained by simply the natural variability which exists between clinical decisions made by two professionals.

Session durations, however, were slightly longer than a traditional FTF assessment. On average, the assessment took three quarters of an hour, though this was shorter for less complex patients (who completed tasks more quickly and required fewer food/fluid trials). Considering the time needed to change/adjust the equipment during the session, the need for interaction between the online clinician and assistant, occasional technical difficulties, and the need to orientate patients to the online session, this slightly increased session duration is to be expected. It is also not excessively beyond the 20–30 minutes typically taken, in our clinical experience, to complete a thorough FTF CSE session. The 4-hour clinic model was also easily incorporated within the departments' speech pathology service. Following the format of other specialist speech pathology clinics (e.g., an instrumental dysphagia assessment clinic), having a routine, weekly clinic, helped with the organization of staff, equipment, rooms, and referrals.

Patient perceptions of the clinical service were positive and 98% felt comfortable receiving services via telerehabilitation to assess their swallowing disorder. Although prior to the session there was a proportion of patients who were unsure about some aspects of the service (e.g., visual or auditory quality), significantly less were concerned about these issues after the session. Indeed, as has been observed in other clinical groups [12, 14, 15], across all questions there was a shift toward even greater acceptance of the telerehabilitation modality after just one session. However, it was noted that whilst 99% felt comfortable with their assessment, 37% indicated they would prefer a traditional consultation. A number of studies have found similar results, with a small proportion of patients having a preference for traditional clinical models [16–18]. Although reasons for this decision were not examined, there is growing interest in exploring the demographics of users of various types of teleservices [19], and this is an area of future research.

Clinicians were positive about the clinic and felt satisfied with the levels of service and patient rapport established in over 90% of sessions. Whilst certain patient characteristics were noted which enhanced the complexity of assessing a patient in the online environment, clinical decisions were achieved for all patients and the quality of decision making remained comparable to the FTF clinician. This finding is consistent with previous studies which have noted that assessing patients of greater severity may be more complex, but is not impossible to achieve via telerehabilitation [3, 13]. Key factors that clinicians felt contributed to the success of the sessions and the clinic overall included the importance of appropriate referrals, sufficient clinical notes about each patient to enable adequate preparation (particularly for more complex patients), having a team of trained staff, and clear procedures.

## 5. Conclusion

The current trial provided valuable insight into the service issues associated with implementing a dysphagia assessment clinic via telerehabilitation. Overall the clinic was found to run with acceptable efficiency, with minimal technical difficulty, and with comparable primary patient outcomes to FTF assessment. Patient satisfaction was high and clinicians were satisfied with the service in over 90% of the assessments conducted. It is acknowledged, however, that this was a short-term trial clinic and used a simulated service model. Future studies of actual remote clinical implementation will enable exploration of additional factors such as changes to waiting time for services, impact on clinician and/or patient travel time, and evaluation of the economic cost-benefits of service models within various different clinical contexts (e.g., outpatient services, residential aged care services) [20].

## Figures and Tables

**Table 1:** Pre- and postassessment patient questionnaire (*n* = 82).

Item	Preassessment			Postassessment				
	Strongly disagree/disagree *n* (%)	Unsure *n* (%)	Strongly agree/agree *n* (%)	Strongly disagree/disagree *n* (%)	Unsure *n* (%)	Strongly agree/agree *n* (%)	*X* ^2^ or fisher	*P *
(1) I will be *(am#)* comfortable to use telehealth if it is available in the hospital or healthcare facility nearest to my place of residence.	1 (1)	8 (10)	73 (89)	0 (0)	2 (2)	80 (98)	3.78	0.209
(2) I am *(was)* comfortable to undergo an assessment for my swallowing disorder via the internet.	3 (4)	3 (4)	76 (93)	0 (0)	1 (1)	81 (99)	0.08	1.000
(3) I will be *(was)* comfortable being online and would consider using the internet for the rehabilitation of my swallowing.	1 (1)	12 (15)	69 (84)	0 (0)	3 (4)	79 (96)	6.75	0.090
(4) I will have *(I had)* no difficulty in seeing the online speech pathologist.	26 (32)	26 (32)	30 (37)	15 (18)	1 (1)	66 (80)	11.38	**0.008**
(5) I will have *(I had)* no difficulty hearing the online speech pathologist.	23 (28)	27 (33)	32 (39)	19 (23)	3 (4)	60 (73)	3.00	0.601
(6) I would rate the online assessment as being equal to an assessment conducted traditionally in the face-to-face method.	4 (5)	31 (38)	47 (57)	3 (4)	11 (13)	68 (83)	11.66	0.030
(7) The instructions during the online assessment will be *(were)* clear and easy to follow.	1 (1)	26 (32)	55 (67)	2 (2)	3 (4)	77 (94)	7.13	0.075
(8) I will have *(had)* sufficient time to execute the instructions given during the assessment.	2 (2)	25 (30)	55 (67)	0 (0)	1 (1)	81 (99)	2.31	0.329
(9) I will have *(had)* opportunities to clarify any doubts during the online assessment.	0 (0)^a^	14 (17)	67 (88)	0 (0)	0 (0)	82 (100)	n/a^b^	n/a^b^
(10) Telehealth can replace a face-to-face assessment.	10 (12)	25 (30)	47 (57)	7 (9)	18 (22)	57 (70)	36.06	**<0.005**
(11) Telehealth will allow easy access to healthcare.	0 (0)	11 (13)	71 (87)	0 (0)	7 (9)	75 (91)	5.71	**0.047**
(12) Telehealth will save me travelling time and money.	2 (2)^ a^	5 (6)	74 (91)	0 (0)	3 (4)	79 (96)	12.42	0.082
(13) Telehealth may benefit all patients alike.	1 (1)^ a^	19 (23)	61 (75)	2 (2)	8 (10)	72 (88)	15.04	**0.003**
(14) I would prefer to have a traditional consultation with the speech pathologist despite possible costs and inconveniences.	19 (23)^ a^	30 (37)	32 (40)	33 (40)	19 (23)	30 (37)	29.29	**<0.005**

Note: #: italics and brackets indicate pre- /postwording changes between the pre- and postassessment conditions. ^a^ *n* = 81; ^b^not enough variation in the data to complete statistical analysis.

Bold indicates significance at *P* < 0.05.

**Table 2:** Clinician perceptions of the telehealth session (*n* = 100).

	Strongly disagree (%)	Disagree (%)	Unsure (%)	Agree (%)	Strongly agree (%)
I was satisfied with the level of service the computer system allowed me to provide my clients	0	7	1	57	35
I am happy with the level of client-clinician rapport generated during this session	0	4	4	42	50
I found the computer and computer system easy to use during the session	0	4	1	41	54
The audio quality of the system was appropriate for the session	1	14	3	44	38
The visual quality of the system was appropriate for the assessments performed	0	12	5	65	18
I feel that I was able to satisfactorily and competently assess the client to the best of my abilities using the system	1	9	3	46	41
I feel that the telerehabilitation system would be a more efficient means of service delivery for this patient	0	12	12	23	53
I feel the telerehabilitation system would be a useful service delivery tool for patients with swallowing disorders	0	0	2	21	77
